# The COVID-19 pandemic: a case for epistemic pluralism in public health policy

**DOI:** 10.1007/s40656-020-00353-8

**Published:** 2020-12-14

**Authors:** Simon Lohse, Karim Bschir

**Affiliations:** 1 Centre for Ethics and Law in the Life Sciences (CELLS) and Institute of Philosophy, Leibniz University Hannover, Hannover, Germany; 2 School of Humanities and Social Sciences, University of St. Gallen, St. Gallen, Switzerland

**Keywords:** Evidence-based policy, Paul Feyerabend, Expertise

## Abstract

This paper uses the example of the COVID-19 pandemic to analyse the danger associated with insufficient epistemic pluralism in evidence-based public health policy. Drawing on certain elements in Paul Feyerabend’s political philosophy of science, it discusses reasons for implementing more pluralism as well as challenges to be tackled on the way forward.

We live in an age of evidence-based policy. Recent attacks on science in the US, Germany and other countries and greater public scrutiny—sometimes leading to outright denial—of scientific knowledge are no contradiction to this claim. Rather, sceptical and anti-science movements must be understood against the backdrop that science today is much more *relevant* to politics, public life, and the economy than it ever was before.^1^

The significance of science for policy-making was hardly ever more visible than during the COVID-19 pandemic in 2020. Scientific experts informed and drove governmental crisis management and public health policy strategies in many countries and on many levels. In the US, the UK and the EU, for example, epidemiological models and scientific advice had a direct influence on the implementation of lock-down measures to prevent disease spread and on the development of strategies to deal with the pandemic on a long-term basis (e.g. Adam 2020).

There have been two main lines of criticism about the way science informed and guided policy during the COVID-19 pandemic. The first type of criticism concerns the fact that government action has been mainly driven by numbers of COVID-19 cases and deaths at the expense of other aspects of the situation. For example, Caduff (2020) claims that many governments failed to take into account the drastic impact of lock-down measures on the economy and the lives of less affluent citizens. It has also been argued that social consequences of lockdowns, such as the risk of increased domestic violence or the amplification of social inequalities due to home schooling, were not adequately taken into account.

The second type of criticism concerns in silico modelling and its central role in strategic policy decisions. It has been pointed out that policy-making relied far too much on epidemiological modelling despite the fact that even the best computer models suffer from severe uncertainty and that they may be based on misleading mathematical simplifications (Saltelli et al. 2020). Computer models can help to project possible scenarios. But they often contain estimations of initial conditions and assumptions about the dynamics of a system which are made under uncertainty. Uncertainties in the initial conditions or the dynamical structure of the model may translate to the projections derived from these models. This effect is especially pronounced in non-linear models. The amplification of structural model errors in predictions has been described by Frigg et al. (2014) as hawkmoth effect (in analogy to the butterfly effect that arises due to uncertainty in the initial conditions). The hawkmoth effect may seriously compromise the usefulness of nonlinear models for predictive purposes.^2^ As a consequence, policy-making may have been guided by only apparently exact projections of the effects of certain policy measures while not paying enough attention to alternative information for policy-making, in particular information regarding different impacts of policy measures in different socio-economic sectors of society.

While both types of criticism address different issues, they share a concern about insufficient epistemic pluralism in the public health measures that were implemented to address the COVID-19 pandemic. Generally speaking, epistemic pluralism is the use of more than one perspective or approach to deal with a knowledge-related problem. We provide a short analysis of the problems arising from *insufficient* epistemic pluralism in a public health context that is inspired by certain elements in Paul Feyerabend’s political philosophy of science. The goal is to deepen our understanding of potential epistemic shortcomings of evidence-based public health policy and to provide part of a multi-layered answer to a key question in this context: *How should we design future evidence*-*based policy-making in the context of public health threats?*

The diagnosis of insufficient epistemic pluralism is rather straightforward in both lines of criticism mentioned above. In both cases, policy-making seems to have been constrained in that it mainly focused on *one* epistemic perspective or approach. According to the first line of criticism, public health policy was developed primarily from a biomedical perspective at the expense of alternative viewpoints, e.g. viewpoints based on local knowledge or non-scientific forms of knowledge. According to the second line, policy making was constrained by epidemiological modelling ignoring relevant perspectives form other biomedical disciplines and the social sciences. The first type of criticism mainly concerns a lack of pluralism regarding viewpoints that are external to science, while the second type concerns a lack of pluralism within science itself. Both forms of insufficient pluralism—external and internal to science—are problematic from an epistemological point of view.^3^

*First*, there is a risk of developing a myopic, epidemiology-centric description of reality that can lead to imbalanced policy decisions. For instance, the strong focus on the numbers of COVID-19 cases and deaths may have led to one-sided harm-benefit analyses that only addressed COVID-19-related health threats while disregarding potential and/or latent social issues that have been consequences of pandemic crisis management. Likewise, the overreliance on Susceptible-Infected-Recovered (SRI) models may have structurally excluded potentially useful knowledge from other biological disciplines or the social sciences, knowledge that could have been crucial for more rational policy interventions (Manzo 2020).

*Second*, we have limited knowledge about pandemics and their development in general. But even if this were the case, there are always good reasons to take a fallibilistic stance towards our current best scientific knowledge. Disease statistics can always misrepresent, and models can always be wrong. We should thus keep our options open and promote the development and use of alternative ways to understanding what is going on. In Feyerabend’s words: “[T]he world which we want to explore is a largely unknown entity. We must, therefore, keep our options open and we must not restrict ourselves in advance” (Feyerabend 1993[1975], p. 12).

*Third,* and in line with another key argument by Feyerabend (1999[1968]), it is always rational to develop alternative approaches to any given epistemic problem to better understand the shortcomings of each alternative. It is only when one has an independent perspective on the local side-effects of policy-measures that deal with the COVID-19 pandemic that one can see what (else) might be missing from the picture of a specific public health strategy, and insights and methodological approaches from social scientists can be vital in identifying misguided assumptions in the predictions of epidemiological models.

What follows from the observed lack of epistemic pluralism in the management of the COVID-19 crisis? It seems that the right move would be to strengthen epistemic pluralism in evidence-based public health policy. Many more perspectives should be included in providing the evidence for policy-making and many more stakeholders should get a voice in policy-counselling (instead of criticizing policy-making from the outside). This would include not only epidemiologists, social scientists and scholars from other fields but also experts who could contribute local knowledge of relevant social spheres, such as nurses or education department heads who know which hygiene measures can realistically be implemented in their primary schools.

It is important to note, however, that most of Feyerabend’s arguments for pluralism are negative in the sense that they are directed *against* all forms of epistemic monism and methodological hegemony. Feyerabend is less pronounced when it comes to developing a constructive and positive account of how pluralism can be implemented in practice. In fact, the promotion of epistemic pluralism is fraught with challenges. Among the most pressing are the following three: (a) There are pragmatic constraints on the inclusion of more, diverse perspectives and types of evidence in policy-making, especially in times of acute public health threats. How many perspectives can be included without completely paralyzing the policy process? (b) Exchange between diverse perspectives has to be facilitated while avoiding the inclusion of what Feyerabend has called “cranks” (see the discussion in Shaw 2020), i.e. stakeholders who are only interested in defending their own point of view or interests, and not in the open exchange of ideas and in learning from each other (think “conspiracy theorists”). (c) Differences in epistemic standards and authority have to be overcome. Everybody who has ever worked in an inter- or transdisciplinary environment knows how challenging it can be to discuss different methods, findings and other types of knowledge when epistemic standards are divergent. We should also expect differences in authority (and prestige) between policy experts, scientists and experts on local knowledge. Those may lead to imbalanced discussions, instead of genuine dialogue, and need to be alleviated by actively designing deliberative processes in order to facilitate *a productive form* of pluralistic exchange at eye level (Barker and Kitcher 2014, p. 155ff).

Addressing the issue of insufficient epistemic pluralism as well as the sketched challenges associated with pluralism is essential for improving evidence-based public health policy, in particular in the context of acute public health threats. Both aspects should therefore be on the agenda of scholars who think about evidence-for-use and normative issues in scientific expertise in the context of policy-making.

## Footnotes

1 Although we think that the rise of evidence-based policy, in particular within the last third of the twentieth century, is an important background condition for current science denialism, this is not meant to deny the importance of other, industry-driven factors that actively induce anti-science tendencies, for example by “manufacturing doubt” (Oreskes and Conway 2010).

2 Frigg et al. discuss the *hawkmoth effect* in the context of climate modeling. However, the authors also provide arguments for the applicability of the effect to other modeling contexts that operate with nonlinear models.

3 We wish to thank Thomas Reydon for his helpful comments and for encouraging us to further develop the plea for more pluralism in public health policy, which will be done in a forthcoming publication on the topic.
